# Supplementary material for: Causal Relationship between Aging and Anorexia Nervosa: A White-Matter-Microstructure-Mediated Mendelian Randomization Analysis
Source: Biomedicines. 2024 Aug 16;12(8):1874. doi: 10.3390/biomedicines12081874 (PMC11351342; doi:10.3390/biomedicines12081874)
Supplement: Supplementary file 1 [file biomedicines-12-01874-s001.zip › Supplementary Figure/Figure S4/Singe SNP plot of MR analysis for TL to ubm-b-1537.pdf]

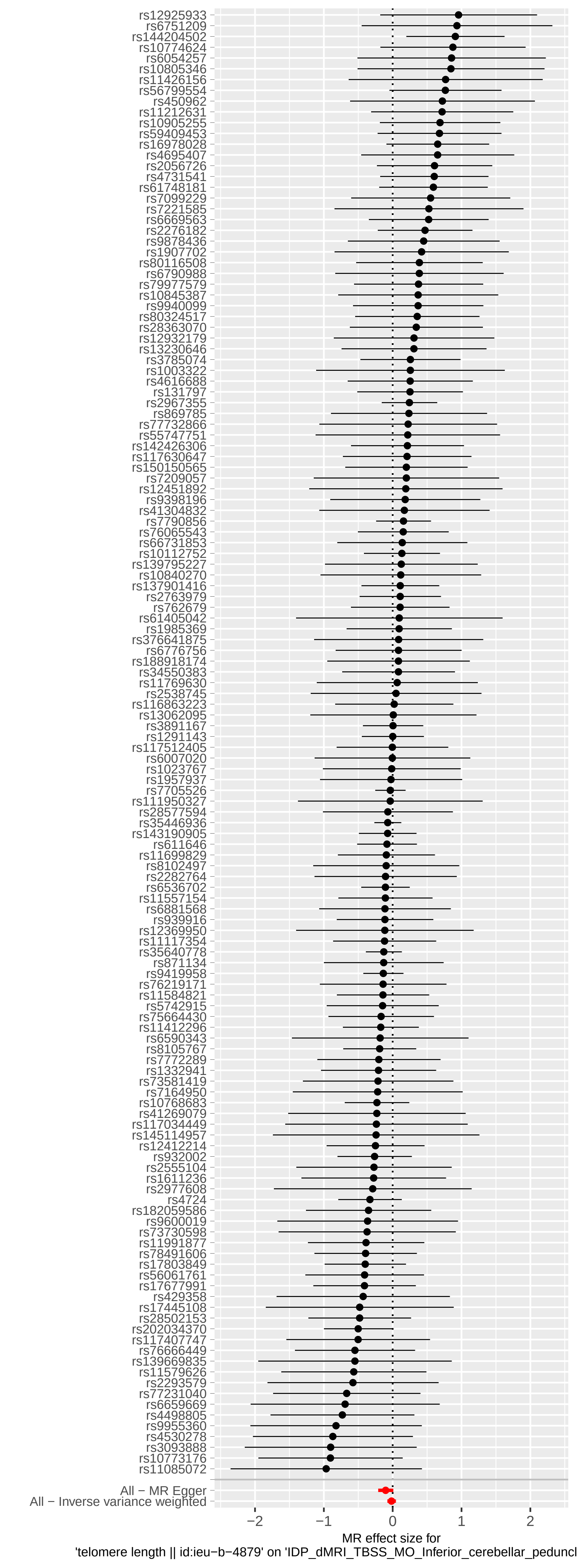
rs12925933
rs6751209
rs144204502
rs10774624
rs6054257
rs10805346
rs11426156
rs56799554
rs450962
rs11212631
rs10905255
rs59409453
rs16978028
rs4695407
rs2056726
rs4731541
rs61748181
rs7099229
rs7221585
rs6669563
rs2276182
rs9878436
rs1907702
rs80116508
rs6790988
rs79977579
rs10845387
rs9940099
rs80324517
rs28363070
rs12932179
rs13230646
rs3785074
rs1003322
rs4616688
rs131797
rs2967355
rs869785
rs77732866
rs55747751
rs142426306
rs117630647
rs150150565
rs7209057
rs12451892
rs9398196
rs41304832
rs7790856
rs76065543
rs66731853
rs10112752
rs139795227
rs10840270
rs137901416
rs2763979
rs762679
rs61405042
rs1985369
rs376641875
rs6776756
rs188918174
rs34550383
rs11769630
rs2538745
rs116863223
rs13062095
rs3891167
rs1291143
rs117512405
rs6007020
rs1023767
rs1957937
rs7705526
rs111950327
rs28577594
rs35446936
rs143190905
rs611646
rs11699829
rs8102497
rs2282764
rs6536702
rs11557154
rs6881568
rs939916
rs12369950
rs11117354
rs35640778
rs871134
rs9419958
rs76219171
rs11584821
rs5742915
rs75664430
rs11412296
rs6590343
rs8105767
rs7772289
rs1332941
rs73581419
rs7164950
rs10768683
rs41269079
rs117034449
rs145114957
rs12412214
rs932002
rs2555104
rs1611236
rs2977608
rs4724
rs182059586
rs9600019
rs73730598
rs11991877
rs78491606
rs17803849
rs56061761
rs17677991
rs429358
rs17445108
rs28502153
rs202034370
rs117407747
rs76666449
rs139669835
rs11579626
rs2293579
rs77231040
rs6659669
rs4498805
rs9955360
rs4530278
rs3093888
rs10773176
rs11085072
All − MR Egger
All − Inverse variance weighted
−2
−1
0
1
2
MR effect size for
'telomere length || id:ieu−b−4879' on 'IDP_dMRI_TBSS_MO_Inferior_cerebellar_peduncl
